# Exploring Patient and Provider Narratives: A Qualitative Study Identifying Barriers in Liver Transplantation Referral

**DOI:** 10.21203/rs.3.rs-8263977/v1

**Published:** 2025-12-16

**Authors:** Samantha L. Ky, Marie Nunez Duarte, Nicole Garcia, Alexandra T. Hughes-Wegner, Marwan S. Ghabril, Lauren D. Nephew

**Affiliations:** Indiana University; New York Medical College; Indiana University; Indiana University School of Public Health- Bloomington. Bloomington; Indiana University; Indiana University

**Keywords:** Liver Transplantation, Barriers, Referral, Support Systems, Transportation, Substance Misuse, Access, Knowledge

## Abstract

**Background:**

Chronic liver disease represents a significant public health burden in the United States. Liver transplantation (LT) is the only life-saving option for patients with decompensated cirrhosis. Receiving transplantation is a multi-step process, beginning with referral. Barriers to CLD care and transplantation disproportionately affect medically underserved populations, including racial and ethnic minorities and individuals of low socioeconomic status. This study expands the limited research into liver transplantation referrals to identify systemic, interpersonal, and informational barriers.

**Methods:**

Expanding the limited research into barriers to liver transplantation referrals, this study examines experiences from both patients and providers. Semi-structured interviews of 15 patients and 11 providers across 10 different community gastroenterology practices in Indiana occurred and were transcribed verbatim. Data was analyzed using a six-phase framework for thematic analysis, conducted via HyperRESEARCH 4.5.2.

**Results:**

Patient participants were majority male (n = 10) with five female participants, and an age range from 31–71 years ($\overset{-}{\text{x}}$ =55.3, SD = 13.1). Provider participants were comprised of eight physicians, two nurse practitioners, and one physician assistant. All providers practiced specialties related to general GI or advanced GI care. Themes identify barriers such as long travel distances, appointment wait times, system communication challenges, limitations in provider tools for referral, and lack of support resources for patients.

**Conclusions:**

Addressing these barriers may expand liver transplantation referrals, especially among medically underserved communities and marginalized populations.

## INTRODUCTION

Chronic liver disease (CLD), including cirrhosis and hepatocellular carcinoma, represents a significant public health burden in the United States.(1, 2) Cirrhosis is the final, irreversible stage of CLD and can lead to life-threatening complications such as ascites, variceal bleeding, hepatic encephalopathy, and liver cancer.(3) CLD and cirrhosis accounted for 52,222 deaths in 2023 alone, ranking as the tenth leading cause of mortality in the U.S.(4) Liver transplantation (LT) is the only life-saving option for patients with decompensated cirrhosis.(5) However, LT is a long, multi-step process starting with a referral for transplant evaluation. Only after referral can patients be assessed, registered with the United Network for Organ Sharing, and placed on the transplant waitlist. Despite society guidelines outlining indications for LT referral, a study in a safety-net hospital (SNH) setting found only one-third of eligible patients with hepatocellular carcinoma were referred, 5% were waitlisted, and just 1% ultimately received LT.(6) These findings illustrate critical failures in the early stages of the transplant pathway and underscore the importance of timely and appropriate referrals.

Barriers to CLD care and transplantation disproportionately affect medically underserved populations, including racial and ethnic minorities and individuals of low socioeconomic status.(6–9) SNHs and community gastroenterology (GI) practices often serve these populations yet face substantial structural and resource-related challenges.(8) Notably, a third of all cirrhosis-related hospitalizations occur in SNHs. Within SNHs, Black and Hispanic patients having higher incidences of hospitalization for cirrhosis and Black patients experience higher in-hospital mortality.(5) Several counties in Indiana have been identified as medically underserved, with many being rural in nature and having large black or Hispanic populations.(10) Often, utilizing SNH’s more frequently than more affluent areas, giving them higher risk for GI related issues due to delayed care, pointing to large disparities in CLD care in Indiana.(8) Despite disparities at each stage in the transplant process, the referral step represents a key leverage point for intervention to mitigate barriers in access. Referral practices vary depending on the size, location, and resource capacity of each community.(11) Addressing early barriers to referral are essential for improving outcomes and ensuring equitable access to care. Understanding the multilevel barriers to LT referral requires examining both patient and provider perspectives.

Existing studies narrowly focus on outcomes or administrative data, often excluding the lived experiences and nuanced perspectives influencing decision-making in practice. Few interventions to date have adopted a truly multilevel approach. There are only two known interventions targeting LT referral, and neither includes provider and patient voices together.(5, 12) Informed by the National Institute on Minority Health and Health Disparities (NIMHD) research framework and grounded in implementation science, this study aims to explore barriers at the patient, provider, and system levels. (13) This approach mirrors the success of the Southeastern Kidney Transplant Coalition’s RaDIANT initiative, which improved kidney transplant referrals by targeting multiple system levels and engaging affected communities.^12,13^ Like RaDIANT, this study design emphasizes partnership, capacity building, and data-informed decision-making to inform future intervention development. In doing so, it aligns with national goals to eliminate health disparities and improve access to quality care for vulnerable populations.

The purpose of this study is to explore and identify barriers to providing and receiving liver disease care from both provider and patient perspectives. Specifically, it aims to (1) compare and contrast patient and provider insights, (2) identify systemic, interpersonal, and informational barriers; and (3) generate practical strategies to enhance referral practices in community GI settings.

## METHODS

Researchers utilized qualitative methodology to explore the lived experiences of CLD patients and providers treating CLD. The dual perspective of both patients and providers within LT has yet to be explored by current research. Therefore, qualitative methodology is ideal because it allows researchers to identify, using in-depth analysis, relevant personal and contextual factors potentially not brought up otherwise.(14) Qualitative methods, such as thematic analysis, allow for the participants’ voices and perspectives to remain unaltered and strong through the data analysis process. All procedures were conducted in accordance with the Declarations of Helsinki and Istanbul.

### Participant Recruitment

#### Patients.

Eligible participants were Indiana residents aged 18 who were referred to the senior authors institution for LT from any community practice with cirrhosis or a complication of liver disease including HCC. Participants were identified through electronic medical records (EMR), and their clinicians were first contacted through the EMR for confirmation of fit for the study. Then, patients were approached in-person, and a follow-up email was sent to gauge interest. Interested participants were directed to a website, which contained additional study and contact information. Electronic informed consent was obtained before the start of interviews, including permission for audio-recording.

#### Providers.

Eligible providerswere those practicing in Indiana who care for patients with liver disease. Recruitment occurred from January 2024 to August 2024 through email, the Indiana CTSI mailing list, and peer referrals. Clinicians completed a screening questionnaire and demographic survey. Informed consent, including audio recording permission, was obtained electronically and verbally prior to interviews.

### Interviews

A semi-structured interview guide was created for greater flexibility and to encourage new or relevant conversations for providers and patients. Researchers developed interview guides to capture a thorough understanding of the multilevel factors associated with healthcare for patients with liver disease. Each interview guide was developed under the senior author’s direction, informed by their clinical experience treating patients with liver disease and by relevant literature. Primary interviews and secondary probes were designed to encourage deeper conversation. Sessions began with general questions to build rapport, followed by questions related to healthcare access, liver disease diagnosis and treatment, and the resulting psychosocial impact. Interview guides are detailed in the supplementary material. Interviews lasted approximately 60 minutes and were audio-recorded for accuracy during data analysis. Following interview completion, participants completed a brief demographic survey and received a $100 gift card incentive.

### Analysis

We followed Braun and Clarke’s six-phase framework for thematic analysis to guide our data interpretation process.(15) After the verbatim transcription of interviews, the research team engaged in repeated, in-depth readings of the data to establish a shared familiarity with the content, ensuring consistency across team members. To build the codebook, we adopted a combined deductive and inductive strategy, enabling us to remain grounded in both the original interview questions and emergent patterns from the transcripts themselves. The codebook was informed by incorporating constructs from interview guide, preliminary transcript reviews, and relevant literature. Coding was conducted iteratively using HyperRESEARCH 4.5.2, and analysis continued until thematic saturation was achieve.(15) Code development and application were primarily conducted by research assistants, with regular weekly meetings to monitor coding consistency and maintain interrater reliability. Once coding was finalized, we exported the dataset and grouped the codes into broader thematic categories and subcategories. Relevant excerpts were organized to support the thematic construction.(15)

In the final analytic phase, themes were carefully refined to ensure they accurately captured the core meanings present in the data.(15) Primary themes represented broad conceptual patterns, while subthemes (second-order themes) provided additional structure and specificity.(15) The research team convened monthly to assess progress, reflect on the analytic process, and engage in peer debriefing to promote analytical rigor.(16) Ultimately, themes were solidified through team consensus and continuous engagement with the data to ensure coherence with the participants’ overall experiences. All research protocols and procedures were approved by the senior author’s Institutional Review Board.

## RESULTS

### Participant Demographics

#### Patients

The final sample included 15 patients from 10 different clinical sites across Indiana. A majority were male (n = 10) compared to females (n = 5) with an age range from 31–71 years ($\overset{-}{\text{x}}$ =55.3, SD = 13.1). Most participants identified as White/Caucasian (n = 11), while 4 identified as Black/African American. Full patient demographics in Table 1. Additional exemplar quotes from patients in Table 3.

#### Providers

The final sample included 11 providers with 8 physicians, 2 nurse practitioners, and 1 physician assistant from 8 different practices across Indiana. There were 7 male providers and 5 female providers interviewed with in age range of 37–65 years ($\overset{-}{\text{x}}$ =43.7, SD = 9.89) All providers had specialties within General GI or advanced GI care. A majority practiced in urban areas (n = 6), with the remainder in suburban (n = 3) or rural (n = 2) areas. Full provider demographics in Table 2. Additional exemplar quotes from providers in Table 4.

### Patient Theme 1: Physical & Emotional Barriers Timing, Distance, & Transportation

Participants were quick to identify barriers and obstacles they encountered when seeking care and referrals for their liver disease. Difficulties obtaining timely appointments delayed referrals for LT, particularly among patients receiving care from SNHs and community clinics. Many dealt with distance barriers, such as the need to travel out of their country for liver disease care. A few relocated even closer to their healthcare providers to ease the burden of traveling to their appointments. A number of participants also rely on others for transportation, making it difficult to coordinate travel plans for their appointments. One even stated they were removed from the transplant list after being unable to attend their appointments after referral.

Patient #101: “Initially my appointment was gonna be like in September. And I was like I won’t be alive in September”

Patient #115: “I missed a day coming down here and they took me off [of the transplantation list”

#### Emotional & Mental Barriers

Emotional and mental well-being were prominent barriers among participants. Many participants described reluctance to being on the transplant list, stating how overwhelmed they were by the thought of LT. One participant even declined a liver offered to them because they did not feel emotionally prepared to receive the transplant. Some hesitated due to the “burden” they would place on their loved ones because of the extended recovery period after LT.

Participant #108: But when the lady called me, the nurse called me, I just panicked. And long story short, I end up refusing it. But it’s scary”.

Patient #104: “It definitely gives me a lot of anxiety, just the whole, the whole process”

### Patient Theme 2: Patient, Provider, and System Support Patient-Provider Interactions

Most participants described a positive relationship with their GI providers leading up to LT referral. Many stated they felt like active participants in their treatment plans were able to communicate their health priorities with their providers. These priorities included feeling comfortable discussing emotional, mental, financial, and social concerns during their provider interactions. However, some participants expressed discontent with previous providers for how their treatment occurred. One participant explained how their provider seemed dismissive of their concerns and did not take the disease seriously. They attributed these actions to the delay in referral for LT. Another participant stated they wished their provider had referred them sooner to more capable facilities in the area.

Patient #106: I have opened up honestly and fully with all of my providers since then, about my questions, my feelings, you know, as they come, and my needs.

Patient # 101: I wish they would take it more seriously […] You know I’m coming here because there is something wrong.

### Additional Support within the System

Many participants reported social workers had a positive impact on their liver disease care. Those who had utilized their services before stated their helpfulness in navigating the LT referral process and managing their expectations. Social workers and providers often provided educational resources to help patients understand their disease and care. Individuals whose liver disease was related to substance abuse found their social workers and counselors relayed the importance in maintaining their sobriety for LT. Those who had not previously seen a social worker expressed interest in setting up appointments for the duration of their care.

Patient #102: You see social workers and [addiction] counselors. And I’ve learned a lot about that through the counselors and social workers. And they pretty much told me if they catch any alcohol in my system, they’re not going to treat me.

### Patient Theme 3: Overcoming Challenges Patient Resilience

Despite the challenges and uncertainty associated with CLD, most patients demonstrated personal resilience through practicing daily habits, adapting to barriers and maintaining their health. Participants described regularly exercising and eating more nutritious meals to improve their health. Several described their commitment to maintaining their quality of life and fulfilling their societal roles. Many patients reported their ability to continue working full-time and being active participants in their children’s lives despite their CLD. Their consistent description of perseverance demonstrates resilience is an integral part of liver disease treatment.

Patient #102: “I’m probably in as good a shape as I’ve been in 20 years physically, but I have hepatocellular cancer.”

Patient #115: I like to do a lot of yard work, you know, work around the house in my spare time. I like to go fishing”

### Socioemotional Support

Despite their strong resilience, many stated their emotional well-being, and treatment adherence were only achieved with the support of their family, friends, support groups, and religious beliefs. Many used their loved ones to discuss negative thoughts and fears to prepare themselves for LT. Family members and friends were also instrumental for several participants in helping them maintain their lifestyles. They often helped with financial aspects of their treatment, childcare, transportation, and at-home care. Peer support, whether from transplant recipients or fellow patients, was also mentioned as a meaningful source of guidance and encouragement. Many patients mentioned their desire for more people in their situation to utilize support groups, citing the benefits they gained by attending them. In addition to support from close social circles, many participants also drew strength from their faith and religious communities. Participants shared how religion was integral to maintaining hope throughout their treatment.

Patient #105: I talked to my husband. He doesn’t understand a lot, but regardless, I can get it off my chest, and he knows me. He knows me enough to know if something’s bothering me. So I don’t have to, I don’t just say a lot.

Patient #103: I wasn’t told about the support groups until after I was going through the evaluation process for transplant. I feel like they should let all patients in the very beginning..

### Provider Theme 1: Provider-System Barriers System Challenges

Some providers reported challenges in their healthcare systems contributing to delays or barriers in LT referral. One participant stated they do not receive further communication from within their system after the referral is placed. This led to instances where patients are not contacted by their referral center, and the provider is not notified. Providers noticed a lack of resources, limiting the number of patients they could see at their practice, especially within rural communities. This is compounded by a lack of GI physicians who can manage liver disease care, contributing to long appointment wait times for patients further delaying LT referral. Indeed, providers noted their patients often waited several months to see a GI provider who would then refer them for LT. Then, patients wait months to see the provider they were referred to. Providers in in-patient settings commented on a lack of physical spots at transplant centers for patients in immediate need of LT.

Provider #206: If I put a referral in, I don’t really ever hear any feedback or anything like that. It just happens, and then really the only time I know is if the patient responds like, “I have not heard from them. Why is this taking so long?”

Provider #201: I wish that the wait time for patients was not that long, uh, when we report somebody. But that’s not a barrier that is just a problem we all are facing because we have so many patients, we have limited number of resources and providers.

### Lack of Knowledge

Providers acknowledge a lack of knowledge about the referral process and LT. This meant they were unable to explain or answer questions their patients had about the process, sometimes leaving their patients frustrated. Knowledge gaps existed in indications for LT referral. Some providers commented on changing guidelines or standards for LT referral they were unfamiliar with. One provider stated they struggled when patients had comorbidities, where they might be ineligible for LT. No providers described using a standardized tool to help with referral decisions but indicated they would benefit from such tool.

Provider #205: I feel like sometimes I know something is absolute with regards to liver transplant, that this person you would not transplant and with time, those things keep changing.

### Provider Theme 2: Provider Perceived Patient Barriers Social, emotional, and economic barriers

Providers identified similar barriers their patients may encounter to our patient group. Many in community hospitals noted un-insured or underinsured individuals are limited in their liver disease care options because of their financial situations. Those patients may have added difficulty taking time off work to attend appointments, affording treatments, and traveling long distances healthcare centers. Social support was also a concern of many providers for their patients. They stated concerns about referring patients for LT who did not have a strong support system in place to help them succeed after the transplant.

Provider #205: A lot of times they’re in jobs where taking any time off is very difficult. So we hear that and, you know, our clinic access is limited to a couple of days in the week and if you can’t get a day off that day or you can’t, you know, make it up in some other way, or if you work an hourly job and you come to the doctor’s office, well, you may not make rent that month.

Provider #203: Well, there are people that just don’t have the support with them to help them. Like we have patients like that where it’s like, “Well, I don’t think you have consistent [inaudible] to help you through this.”

### Substance Misuse

Substance use among patients was a common concern among providers. Some reported a lack of resources to help individuals struggling with alcohol and called for additional resources to combat alcohol misuse. Most providers indicated they would refer patients with less than six months sobriety, provided they demonstrated intent to stop drinking. However, some worried patients with a history of alcohol misuse would return to drinking after receiving LT and stated they would not refer patients to LT who had not maintained sobriety for several months.

Provider #206: I feel like the resources to help people who have difficulty with alcohol is lacking, not just here, but everywhere. And so sometimes, I feel like that is a big barrier for people if they just either choose not to or are struggling to stop drinking alcohol. It’s a big thing.

Provider #211: I personally wait at least nine months, preferably 12 months, I usually wait nine months because it takes 2–3 months for IU to see them. So by that time they have been sober for about a year, and this is not just being sober.

## DISCUSSION

This study used in-depth qualitative inquiry to present insight into patient and provider experiences with barriers in LT referral. The findings from this research add to the limited number of studies examining barriers to LT referral from the perspective of two important stakeholders.(5, 12)

Many patients described living in communities and environments where it was difficult to attend their LT appointments due to long travel distances, limited transportation, and a lack of available appointments. Moreover, several individuals relied on external means to travel such distances, obtaining rides from family members, friends, or public transit, thereby extending the burden of long travel times to loved ones. Providers validated these transportation barriers, acknowledging the extensive distances many of their patients must travel for care due to the shortage of local providers capable of making or receiving LT referrals. Those practicing in community clinics or safety-net hospitals further emphasized the limited transportation resources available to their patients, many of whom are underinsured or uninsured altogether. These structural limitations exacerbate existing disparities in access to transplant evaluation and follow-up care, placing additional strain on both patients and providers.

Ultimately, social support systems serve a critical role in caring for patients throughout the continuum of LT care due to the emotional and physical toll both before and after transplantation.(17) Chronic liver disease often requires sustained medical management and lifestyle adjustments, further exacerbating stress and healthcare management fatigue when they lack external social support.(17) Many patients highlighted the vital role their family and friends had in navigating their liver disease care. Several participants also described the positive influence of peer and community-based support groups on their mental well-being. Support groups were characterized as safe spaces where patients could share fears, worries, and hopes about transplantation with others who understood their experiences. While the benefits of support groups have been well documented among other transplant populations, limited research has explored their specific impact among LT patients.(18, 19) In this study, these peer-centered conversations emerged as a prominent facilitator of emotional resilience and readiness for transplantation.

Similarly, patients also expressed their interest in utilizing social worker services to help them navigate the complex LT referral process, which they found difficult to navigate leading to delays. Our findings show that it may be beneficial to involve social workers in CLD care prior to referral to providing support and supply resources for patients with substance misuse issues. Social workers are also able to help address the social determinants of health that may prevent a patient from obtaining LT referral. Those who had seen a social worker noted they obtained valuable information about LT, addressing knowledge barriers that would have made referral more difficult otherwise. Providers were also sources of education for patients. Many patients noted their patient-provider relationship as facilitators to their care. Positive experiences included feeling comfort in sharing health priorities such as financial, emotional, and mental concerns about their healthcare with their provider. Inversely, patients who reported negative relationships with their providers felt this was detrimental to their care and delayed their referral to LT.

Patients appreciated when providers were knowledgeable about the LT referral and could provide them with important information about the process. Despite this, many of the providers interviewed admitted they had limited knowledge about LT. Providers stated they usually referenced a patient’s MELD score as a determining factor, but patients with co-morbidities made it difficult to know when to refer. Providers also expressed interest in a standardized tool to help decide whether a patient should be referred for LT. Moreover, the previous standard that patients observed 6 months of sobriety has been largely eliminated as other biomarkers have become better predictors of relapse after transplantation.(20, 21) Despite these studies, some providers still expressed hesitance to refer patients with less than 6 months of sobriety. This lack of knowledge in indications for LT serves as a barrier in referral that should be addressed. Especially, among communities that largely serve marginalized populations as they are most affected by unequal access to transplantation.(9, 22)

Furthermore, many providers acknowledge the lack of resources available to patients with a history of substance misuse. Patients in need of LT whose etiology of liver disease is alcohol misuse often experience greater mental health difficulties and require additional support to maintain their sobriety.(23) However, these individuals often receive less social support from their family and friends than other types of LT patients.(17) This highlights the need to bridge the gap in social support with resources in the healthcare system and LT referral process to better equip these patients in succeeding with their LT.

Challenges within the healthcare system were noted by some providers as stated they don’t ever receive confirmation or feedback after the initial referral has been made. Therefore, if any issues arise that could be addressed by the referring provider, they are not contacted. Patients communicated similar grievances, saying they struggled with referrals to different hospital systems. This indicates a need for healthcare systems to improve communication with all parties involved when managing referrals.

Despite the severity and uncertainty associated with CLD, patients frequently described their determination to preserve and adapt. Many participants demonstrated strong commitment to their health and implemented several life changes such as exercise and healthy eating to achieve their goals, with the support of their loved ones. This resilience in patients plays a powerful role in maintaining their quality of life and societal roles while navigating their CLD. Thus, underscoring the need for future interventions to consider not only the clinical but also the psychosocial dimensions of liver disease care.

### Implications

**Figure 1:**
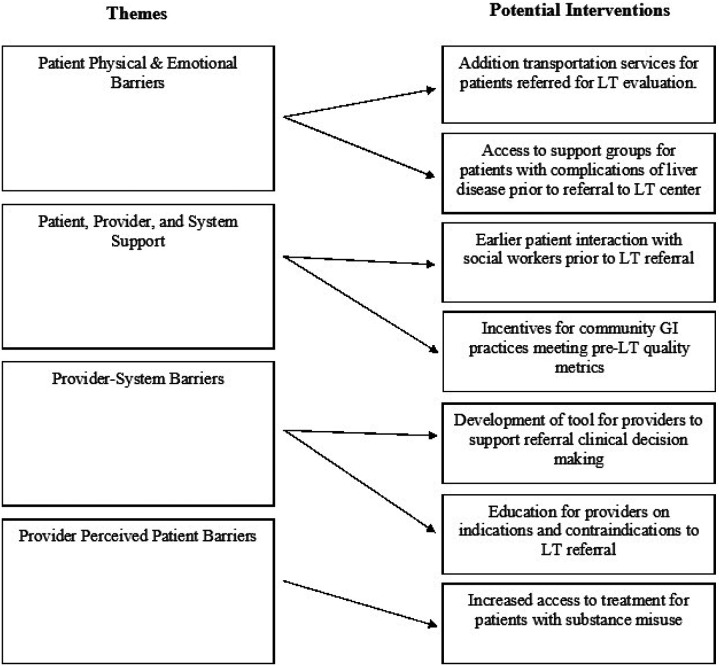
Themes and Potential Interventions

Future efforts to address barriers in LT referral access should target individuals who might experience more transportational and financial difficulties, bridging an existing gap in LT care. Resources should devote incentives for GI specialists and providers to practice in more rural areas, supporting communities with lower access to healthcare. Practices should expand care in these areas with advanced practice providers in collaborative care models to further serve patients by reducing appointment wait times with the few providers currently available. Interventions to increase patient support throughout the LT process should include meetings with social workers, encouraging participation in support groups, and expanding resources for patients with substance misuse to maintain their sobriety. Healthcare systems should prioritize streamlining external and internal processes regarding LT referrals, such that relevant stakeholders remain in contact after the initial referral. Efforts should be made to disseminate referral education to GI providers through short, targeted guidance specific to their practice. Research should focus on developing resources for providers to use when deciding whether a patient should be referred to LT. Full list of potential interventions in Fig. 1.

### Strengths, Limitations, & Future Research

Interviewing both patients and providers allowed for multi-level barriers to emerge during data analysis and interpretation. The diverse sample population included multiple perspectives from participants across different size practices, with a mix of rural, suburban, and urban settings across the state of Indiana. The use of qualitative methods led to a broader understanding of barriers in liver disease referral and patient/provider experiences. This study has certain limitations. While all participants in the study first received their care in a community-based gastroenterology practice, all patient participants ultimately received a referral for liver transplantation. Therefore, there may be other barriers not captured in this study by patients who might have benefited from referral but were not able to obtain one. Additionally, in-person interviewing is subject to recall and social desirability bias that might have skewed participant responses. Our sample size was powered to saturate on themes, yet the non-experimental nature of the study could not establish a causal relationship between the reported barriers and referral ease. Future research includes adapting a clinical decision support tool to facilitate LT referral for providers. These researchers aim to continue educating providers on LT processes and creating accessible alcohol treatment tools to support all CLD patients.

### Conclusion

This study expands the limited research into barriers for LT referrals. By examining experiences from both patients and providers, this research identified personal and system barriers. Challenges included long travel distances, long appointment wait times, limited support for patients with a history of alcohol misuse, limited tools for providers identifying patients in need of LT, and lack of resources of patient support. We call relevant stakeholders to implement interventions to address these barriers and to support patients with chronic liver disease.

## Supplementary Files

This is a list of supplementary files associated with this preprint. Click to download.

- SupplementaryMaterials.docx

## Figures and Tables

**Table 1:** Patient Demographics

Variable		N (%) or $\bar{\text{x}}$ (SD)
Sex	Male	10 (66.7%)
	Female	5 (33.3%)
Race	White/Caucasian	11 (73.3%)
	Black/African American	4 (26.7%)
Ethnicity	Hispanic/Latino	3 (20.0%)
	Non-Hispanic/Latino	12 (80.0%)
Age	n/a	55.2 (13.1)
Primary Insurance	Private Insurance	1 (6.7%)
	Medicaid	9 (60.0%)
	Medicare	5 (33.3%)
Marital Status	Single	8 (53.3%)
	Married	7 (46.7%)
Reported Income	< $15,000	3 (20.0%)
	$15,000-$30,000	2 (13.3%)
	$30,000-$50,000	4 (26.7%)
	$50,000-$75,000	2 (13.3%)
	>$75,000	1 (6.7%)
	Prefer not to answer	3 (20.0%)

**Table 2:** Provider Demographics

Variable		N (%) or $\bar{\text{x}}$ (SD)
Sex	Male	7 (63.6%)
	Female	4 (36.4%)
Age	n/a	43.7 (9.9)
Clinical Setting	Rural	2 (18.2%)
	Urban	6 (54.6%)
	Suburban	3 (27.3%
Provider Type	Physician	8 (72.7%)
	Nurse Practitioner	2 (18.2%)
	Physician Assistant	1 (9.1%)
Years Post Terminal Degree	n/a	10.5 (10.6)
Years in Community	n/a	10.5 (9.6)

**Table 3:** Patient Themes & Exemplar Quotes

Theme	Sub-Theme	Exemplar Quote	ID #
1. Emotional & Physical Barriers	Timing, Distance, & Transportation Barriers	“Initially my appointment was gonna be like in September. And I was like I won’t be alive in September”	101
		“I got to come down here. I got to tell my brother. He got, you know, he got so much time at work that he just used for case of days or whatever, you know, time off”.	115
		“Just the transportation thing has always been, or has become an issue, and I worry about, you know, making sure I have the money and the proper notice to if I got to use medical transport, or if I got to pay for it.”	109
		“I missed a day coming down here and they took me off [the transplantation list]”	115
	Emotional & Mental Barriers	“It definitely gives me a lot of anxiety, just the whole, the whole process”	104
		“But when the lady called me, the nurse called me, I just panicked. And long story short, I end up refusing it. But it’s scary”	108
		“The burden that I’ll be putting on others and things, you know, that would probably be my biggest issue.”	110
2. Patient, Provider, and System Support	Patient-Provider Interactions	“I have opened up honestly and fully with all of my providers since then, about my questions, my feelings, you know, as they come, and my needs.”	106
		“I wish they would take it more seriously. […] You know I’m coming here because there is something wrong.”	101
		“I wish they would have been able to discuss with us better what was going on with me at first, because they were pretty lost with what was going on with me, nobody could figure it out. And I wish they would have reached out to IU, who dealt with livers and who knew more, for help instead of waiting”	103
		“my family’s physician, yeah, a lot said me and him, we could sit down and talk”	115
	Additional Support within the System	“I have also talked to the social worker about, like, my mental health and how it’s overwhelming.”	104
		“You see social workers and [addiction] counselors. And I’ve learned a lot about that through the counselors and social workers. And they pretty much told me if they catch any alcohol in my system, they’re not going to treat me.”	102
3.Overcoming Challenges	Patient Resilience	“I’m probably in as good a shape as I’ve been in 20 years physically, but I have hepatocellular cancer.”	I02
		“Well, overall, I feel pretty good. I have some days where, you know, I’m more tired than others, and I might need to do more resting and just relax and not doing things. Sometimes I do have those things, but overall, I try to, you know, do whatever is required and needed,”	112
		“I like to do a lot of yard work, you know, work around the house in my spare time. I like to go fishing”	115
	Socioemotional Support	“I talked to my husband. He doesn’t understand a lot, but regardless, I can get it off my chest, and he knows me. He knows me enough to know if something’s bothering me. So I don’t have to, I don’t just say a lot”	105
		“I’ve learned a lot in the support group as to what to expect and how I should handle things”	102
		“I wasn’t told about the support groups until after I was going through the evaluation process for transplant. I feel like they should let all patients in the very beginning.	103
		“he would come in and pray for us before we went into surgery. We’d all pray together”	107

**Table 4:** Provider Themes and Exemplar Quotes

Theme	Sub-Theme	Exemplar Quote	ID #
1. Provider – System Barriers	System Challenges	“If I put a referral in, I don’t really ever hear any feedback or anything like that. It just happens, and then really the only time I know is if the patient responds like, ‘I have not heard from them. Why is this taking so long?’”	206
		“I wish that the wait time for patients was not that long, uh, when we report somebody. But, that’s not a barrier that is just a problem we all are facing because we have so many patients, we have limited number of resources and providers.”	201
		“I really didn’t know because it really depends on like if you look at my schedule now, I’m booked until like November, December.”	202
		“There is a lot of patients on the inpatient service that we like for them to be at the transplant center. Uh, but they don’t have beds, so they remain with us and that is I think that is a very major problem they have.”	209
	Lack of Knowledge	“I feel like it’s because I don’t know a lot about the process, honestly. It’s hard for me to answer questions if they do have them, because I don’t know enough about the process.”	206
		“So it’s more that at Eskenazi there is something now that I just don’t know about. At the university hospital, I have no idea because we’re in two different ends of the hospital. So I don’t know what they do in the liver clinic.”	207
		“I feel like sometimes I know something is absolute with regards to liver transplant, that this person you would not transplant and with time, those things keep changing. “	205
2.Provider Perceived Patient Barriers	Social, Emotional, Economic Barriers	“I mean, Eskenazi, as a whole, cater to patients who do not have insurance. I think they have a few social programs in place, which is why we tend to see a higher percentage of that particular patient demographic, but still it does limit them in some ways. Patients, for example, need to be referred to tertiary centers like the university hospital. That’s when it becomes a problem.”	204
		“I would say it’s the fear. It’s thinking they’re going to need a transplant, a major operation and know they’re going to have to take medicine after that, probably for the rest of their lives, that kind of thing. It’s overwhelming.”	202
		“A lot of times they’re in jobs where taking any time off is very difficult. So we hear that and, you know, our clinic access is limited to a couple of days in the week and if you can’t get a day off that day or you can’t, you know, make it up in some other way, or if you work an hourly job and you come to the doctor’s office, well, you may not make rent that month.”	205
		“Well, there are people that just don’t have the support with them to help them. Like we have patients like that where it’s like, “Well, I don’t think you have consistent [inaudible] to help you through this.”	203
	Substance Misuse	“I think there’s also some misconception and I think it’s more predominantly amongst patients who have alcohol-associated liver disease, that once they get a new liver, it’s like a get out of jail free card and they can start drinking again. And I think sometimes we mentioned in passing or, you know, you can’t drink again and they’re fine with it in the moment when they’re still sick. But then when they get a new liver, a lot of them, not a lot, some of them tend to relapse.”	204
		“I feel like the resources to help people who have difficulty with alcohol is lacking, not just here, but everywhere. And so sometimes, I feel like that is a big barrier for people if they just either choose not to or are struggling to stop drinking alcohol. It’s a big thing.”	206
		“I personally wait at least nine months, preferably 12 months, I usually wait nine months because it takes 2–3 months for IU to see them. So by that time they have been sober for about a year, and this is not just being sober.”	211

## Data Availability

Data are available by request from the corresponding author.

## Abbreviations

CLD: Chronic Liver Disease
LT: Liver Transplantation
SNH: Safety-net Hospital
GI: Gastroenterology
EMR: Electronic Medical Records
